# Combined Venom Gland Transcriptomic and Venom Peptidomic Analysis of the Predatory Ant *Odontomachus monticola*

**DOI:** 10.3390/toxins9100323

**Published:** 2017-10-13

**Authors:** Kohei Kazuma, Keiichi Masuko, Katsuhiro Konno, Hidetoshi Inagaki

**Affiliations:** 1 Institute of Natural Medicine, University of Toyama, 2630 Sugitani, Toyama, Toyama 930-0194, Japan; cokazuma@i.nagoya-u.ac.jp (K.K.); kkgon@inm.u-toyama.ac.jp (K.K.); 2 School of Business Administration, Senshu University, 2-1-1 Higashimita, Tama-ku, Kawasaki, Kanagawa 214-8580, Japan; kmasuko@isc.senshu-u.ac.jp; 3 Biomedical Research Institute, National Institute of Advanced Industrial Science and Technology (AIST), 1-1-1 Higashi, Tsukuba, Ibaraki 305-8566, Japan

**Keywords:** *Odontomachus monticola*, venom gland, transcriptome, peptidome, pilosulin-like peptide

## Abstract

Ants (hymenoptera: Formicidae) have adapted to many different environments and have become some of the most prolific and successful insects. To date, 13,258 ant species have been reported. They have been classified into 333 genera and 17 subfamilies. Except for a few Formicinae, Dolichoderinae, and members of other subfamilies, most ant species have a sting with venom. The venoms are composed of formic acid, alkaloids, hydrocarbons, amines, peptides, and proteins. Unlike the venoms of other animals such as snakes and spiders, ant venoms have seldom been analyzed comprehensively, and their compositions are not yet completely known. In this study, we used both transcriptomic and peptidomic analyses to study the composition of the venom produced by the predatory ant species *Odontomachus monticola*. The transcriptome analysis yielded 49,639 contigs, of which 92 encoded toxin-like peptides and proteins with 18,106,338 mapped reads. We identified six pilosulin-like peptides by transcriptomic analysis in the venom gland. Further, we found intact pilosulin-like peptide 1 and truncated pilosulin-like peptides 2 and 3 by peptidomic analysis in the venom. Our findings related to ant venom peptides and proteins may lead the way towards development and application of novel pharmaceutical and biopesticidal resources.

## 1. Introduction

Ants (Formicidae) have many traits in common with social and solitary wasps and bees, including morphology, behavior, and bionomics. Like such Aculeata, most ant species have a sting which they use in both predation and defense. The potency of the venom is attributed to various irritant or paralytic constituents, some of which are potential lead compounds in drug development.

To date, conventional biochemical and molecular biological methods have been employed to identify toxin-like peptides and proteins from several ant species. In our previous studies, we identified bioactive peptides from *Myrmecia* species complex [1,2] and *Strumigenys kumadori* [3] by a PCR-mediated method. In addition, de novo sequencing and transcriptome analysis yielded many toxin-like peptides and proteins from the venom glands of the predatory ants *Dinoponera quadriceps* [4] and *Tetramorium bicarinatum* [5]. Considering the number of ant species already identified, however, the number of toxin-like peptides and proteins which have been identified from ants are so limited thus far.

**Figure 1:**
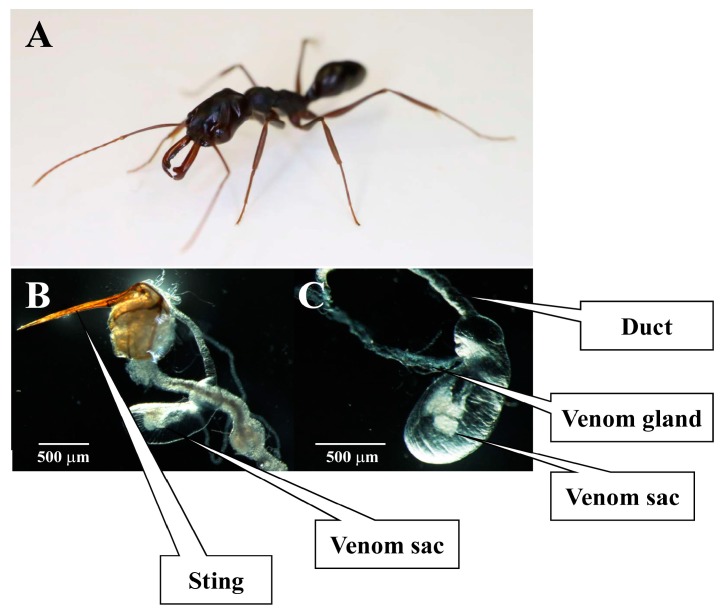
*Odontomachus monticola* venom sting, sac, and gland. *O. monticola* (**A**) and its venom sac and gland were dissected from the abdomen (**B**). Venom sac and gland of panel B were magnified (**C**).

*Odontomachus monticola*, a predatory ant species in the subfamily Ponerinae, is about 10 mm long, red-brown in color, and has long mandibles. *O. monticola* is distributed mainly throughout south- and southeast Asia. In Japan, these ants were originally confined to the southwestern part of the country [6]. Nevertheless, their habitat range has expanded to Tokyo due to climate change over the last few decades. They use their powerful stings to prey upon termites. Although they are not aggressive toward humans, their stings cause intense pain and prolonged itching (Figure 1).

In previous studies, Aili et al. used LC-MS to analyze the venoms of *O. hastatus* in French Guiana and found 528 molecular masses in the venom, 27 of which were disulfide-bonded [7,8]. The primary structures and biological activities of these peptides have not yet been elucidated.

In this study, we used both transcriptomic and peptidomic analyses to identify novel toxic peptides and proteins of *Odontomachus monticola*, a heretofore unexamined species. We identified six pilosulin-like peptides, one chitinase-like protein, one icarapin-like peptide, one hyaluronidase-like protein, four dipeptidyl peptidases, four inhibitor cystine knot (ICK)-like peptides, three phospholipase A_2_-like proteins, and others. In addition, we found certain factors which activate the toxin precursors.

## 2. Results and Discussion

### 2.1. Ilumina Hiseq 2500 Deep RNA Sequencing, Assembly, and Annotation

To characterize the gene expression profile of the *O. monticola* venom gland, we analyzed its mRNA using the Next Generation Sequencer, Illumina Hiseq 2500 and obtained 44,087,058 100-bp reads. The de novo assemblies with Trinity yielded 49,639 contigs (N50 contig 1466 bp). After the highly homologous and duplicated contigs were integrated with cd-hit-est, 43,662 of them were compared against the Uniprot KB database with the BLASTX program in order to determine their potential functions.

About 69.3% of the contigs were found to resemble the transcripts in the Uniprot KB database. We found that two contigs, Om13705_c0_g1_i1 and Om23716_c0_g1_i1, corresponded to 18 s and 28 s rRNAs, respectively. In addition, the 1897 contigs with similarities to bacterial transcripts (Figure S1) might indicate symbiotic microbes in *O. monticola*. Some of these microbes might be utilized for the supply of amino acids and vitamins to the host ant species [9]. These contigs were eliminated from the *O. monticola* transcript data.

To estimate the relative expression levels of the venom gland transcripts, the input reads were mapped to the contigs and counted by Bowtie 2. Five contigs at the top of the read counts resembled an antimicrobial ant peptide, pilosulin, and a toxic honey bee (*Apis mellifera*) peptide, melittin, in predicted amino acid sequences. We named the peptides encoded by these contigs pilosulin-like peptides 2, 3, 4, 5, and 6.

We used PCR in the attempt to find other transcripts encoding pilosulin-like peptides. We designed two 20-base primers corresponding to the leader sequence and the 3′ untranslated region. These are conserved in pilosulin-like peptides 2 and 4. We found another cDNA clone that encodes a pilosulin-like peptide and called it pilosulin-like peptide 1. Pilosulin-like peptides 1, 2, and 4 share conserved 5′ and 3′ untranslated sequences and a leader sequence. Therefore, assembly software could not discriminate among the reads derived from each pilosulin-like peptide, and might not generate a contig coding for pilosulin-like peptide 1. After adding the pilosulin-like peptide 1 sequence to the 41,764 reference sequences and eliminating the rRNA and bacterial transcripts from them, all input reads were mapped to them and counted again by Bowtie 2 in order to estimate the relative venom gland transcript expression levels (Table 1).

**Figure 2:**
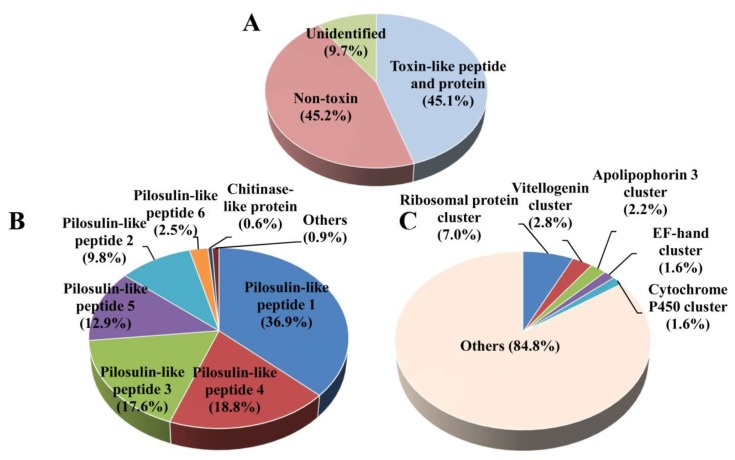
The *Odontomachus monticola* venom gland gene expression profile. The relative expression levels of the genes encoding toxin-like peptides, proteins, non-toxins, and unidentified molecules are displayed as percentages of mapped reads in the total reads (**A**). The distribution of toxin-like peptide- and protein gene expressions (**B**) and non-toxin gene expressions (**C**) are shown. The relative expression levels of the genes are typically reported as RPKM (reads per kilobase of exon model per million mapped reads) in order to correct for the differences in length of individual transcripts. Nevertheless, transcript lengths may not, in fact, influence the expression levels, because those for the abundantly expressed toxin-like peptides and proteins were short.

Ninety-two contigs corresponding to toxin-like peptides and proteins and their isoforms were selected from the database search. The relative expression levels of the toxin-like peptide and protein genes accounted for 45.1% of all reads. Pilosulin-like peptide transcripts constituted 98.5% of the toxin-like peptide and protein reads in the venom transcripts. The remaining 1.5% represented the expression of other toxin-like peptide and protein genes in the venom gland (Figure 2A,B).

Despite the wide range of similarities in the amino acid sequences among individual toxin-like peptides and proteins, the combination of toxin-like peptides and protein transcripts in *O. monticola* venom gland resembled that of *T. bicarinatum* [5]. We found pilosulin-like peptides, ICK-like peptides, phospholipase A_2_-like proteins, waprin-like peptides, hyaluronidase-like proteins, venom allergen 1 (Sol i 1-like protein and phospholipase A_1_-like protein), venom allergen 2/4 (Sol i 2/4), venom serine proteases, and venom carboxylesterase in two species. The venom glands in the two ant species are rich in pilosulin-like peptide transcripts and poor in the rest of the toxin-like peptide and protein transcripts.

Venom gland transcriptomic analyses of some parasitoid wasps (Hymenoptera) have been reported. In contrast to the combination in the two ant species mentioned above, they seem to have few or no linear peptides, like pilosulin and melittin [10,11].

The relative expression level of non-toxin genes accounted for 45.2% of all reads. They were rich in ribosomal proteins (7.0%), vitellogenins (2.8%), apolipophorins (2.2%), elongation factors (1.6%), and cytochrome oxidase P450 (1.6%) (Figure 2A,C).

In the non-toxin and toxin contigs, we found some transcripts which encode the factors that activate or modify the toxins. These include protein disulfide-isomerases (Om23447_c0_g1_i1, Om12259_c2_g2_i2), dipeptidyl peptidases (Om12825_c0_g9_i1, Om4088_c0_g1_i1, Om1574_c0_g1_i1, Om4797_c0_g1_i1), amidating lyases (Om9737_c0_g1_i3, Om1447_c0_g1_i1), and carboxypeptidases (Om9464_c0_g1_i1).

### 2.2. Toxin-Like Peptides and Proteins

#### 2.2.1. Pilosulin-Like Peptides

**Figure 3:**
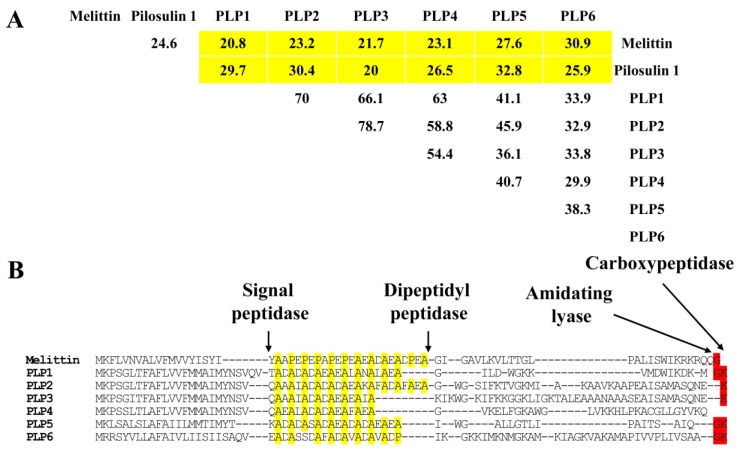
Multiple alignment and identity matrix of pilosulin-related peptide amino acid sequences. Percentage amino acid sequence identities between melittin (NP_001011607.1), pilosulin 1 (Q07932.1), and pilosulin-like peptides (PLPs) are shown in (**A**). The amino acid sequences of melittin and pilosulin-like peptides were aligned with ClustalW in Lasergene 12 (DNASTAR, Madison, WI, USA). Arrows indicate the putative processing and modification sites for signal peptidase, dipeptidyl peptidase, amidating lyase, and carboxypeptidase. Proline and alanine residues in the spacer region between the signal- and mature peptides of pilosulin-related peptides are highlighted in yellow. Nucleotide sequences for PLP 1, 2, 3, 4, 5, and 6 were assigned DDBJ/EMBL/GenBank Accession Numbers LC316119, and FX985495-9, respectively (**B**).

In our previous studies, we identified pilosulins 3, 4, and 5 from *Myrmecia pilosula* species complex [1,2]. Pilosulin-like peptides resemble mellitin and pilosulin in terms of their precursor amino acid sequences (20.0–32.8%) (Figure 3A). In the precursor leader sequences, both pilosulins and pilosulin-like peptides contain a repeated sequence with a proline or alanine residues at every second or fourth position and aspartic or glutamic acid residues between them. Because of the low similarities, we could not find a common feature among pilosulin-like peptides in the mature forms, except for pilosulin-like peptides 1, 2, 3, 4, 6, which are alpha helices, amphiphilic, and basic peptides.

Some of the bioactive linear peptides in Hymenoptera like melittin [12], eumenine mastoparan-OD-like peptide [13], dinoponeratoxin [4], and pilosulin [14] share the same unique repeated sequences in their leader sequences. These repeats might be a common feature of all bioactive linear peptides in Hymenoptera. The repeated sequences could be processed by dipeptidyl peptidase 4 [15], which cleaves the precursor peptides after the proline and alanine residues to yield dipeptides next to an endopeptidase (Figure 3B). In fact, dipeptidylpeptidase 4 (Om4088_c0_g1_i1) was moderately expressed in the venom gland (0.15% of all toxin-like peptide and protein reads).

Many bioactive peptides, which have glycine or glycine-lysine residues at the C-terminus in the precursor, tend to be amidated at this site in the mature forms. Pilosulin-like peptides 1, 5, and 6 have glycine-lysine residues at their C-termini [16]. Carboxypeptidase (Om9464_c0_g1_i1, less than 0.01% of all toxin-like peptide and protein reads) and amidating lyase (Om9737_c0_g1_i3 and Om1447_c0_g1_i1, less than 0.01% of all non-toxin reads) were slightly expressed in the venom gland. The theoretical masses of pilosulin-like peptides 1, 5, and 6 are 2061.090, 1837.071, and 3704.231, respectively. Many bioactive peptides, which have glutamic acid-lysine residues at their C-termini in the precursors, lose the lysine residues in their mature forms. Therefore, pilosulin-like peptides 2 and 3 could also lose their C-terminus lysine residues. The theoretical masses of pilosulin-like peptides 2 and 3 after C-terminus lysine loss are 3361.753 and 4101.241. Pilosulin-like peptide 4 has one cysteine residue and a theoretical mass of 3166.810. In the *Myrmecia pilosula* species complex, pilosulin 5 monomeric peptides with one cysteine residue are connected by a disulfide bridge as homodimers [2]. Using peptidome analysis, we also found a peptide with the same mass as the pilosulin-like peptide 4 dimer in *O. monticola* venom (Table 2). In summary, two pilosulin-like peptide 4 monomers may be joined by a disulfide bridge to form a dimer with a theoretical mass of 6331.625.

#### 2.2.2. Chitinase-Like Protein

**Figure 4:**
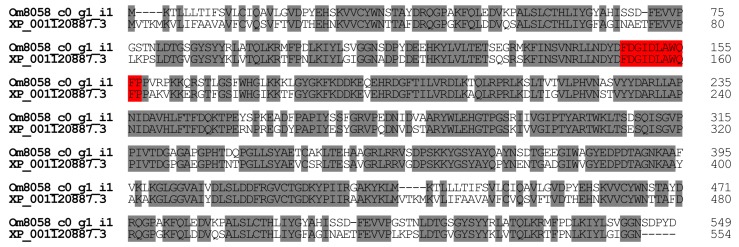
Alignment of chitinase-like protein amino acid sequences. Using ClustalW, the amino acid sequences of chitinase-like proteins were aligned. The catalytic domain and identical amino acid residues were highlighted in red and grey, respectively. The nucleotide sequence of *O. monticola* chitinase-like protein has been assigned DDBJ/EMBL/GenBank Accession Numbers FX985546.

In general, insects use chitinase to digest chitinous materials and to remodel their body structures during ecdysis. Chitinase was detected in the venom gland of an endoparasitic wasp, *Cheonus* spp. In *Cheonus*, chitinase might facilitate the diffusion of the other venom components or the digestion of the prey [17]. A chitinase of the octopus *Eledone cirrhosa* is hemolytic [18]. In *O. monticola*, we found one contig (Om8058_c0_g1_i1) encoding chitinase-like protein (0.51% of all toxin-like peptide and protein reads). *O. monticola* chitinase-like protein displays significant homology (75.7%) with the amino acid sequences of *A. mellifera* (honey bee) chitinase-like protein XP_001120887.3 (Figure 4). Recently, Tetreau et al. [19] classified chitinase-like proteins into eleven groups. On the basis of this system, *O. monticola* chitinase-like protein was placed in group V.

#### 2.2.3. Icarapin-Like Peptide

**Figure 5:**
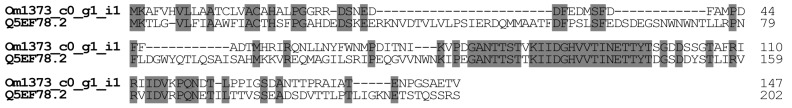
Alignment of icarapin-like peptide amino acid sequences. Using ClustalW, the amino acid sequences of icarapin-like peptides were aligned. Identical amino acid residues of icarapin-like peptides are highlighted in grey. The nucleotide sequence of *O. monticola* icarapin-like peptide has been assigned DDBJ/EMBL/GenBank Accession Numbers FX985505.

We found one contig (Om1373_c0_g1_i1) encoding icarapin-like peptide (0.30% of all toxin-like peptide and protein reads). Icarapin is an IgE-binding *A. mellifera* venom protein. Although recombinant icarapin reacts with sera from humans allergic to bee venom (Api m 10), the primary biological functions of icarapin are unknown [20]. *O. monticola* icarapin-like peptide displays significant homology (49.7%) with the amino acid sequences of *A. mellifera* icarapin Q5EF78.2 (Figure 5).

#### 2.2.4. Hyaluronidase

**Figure 6:**
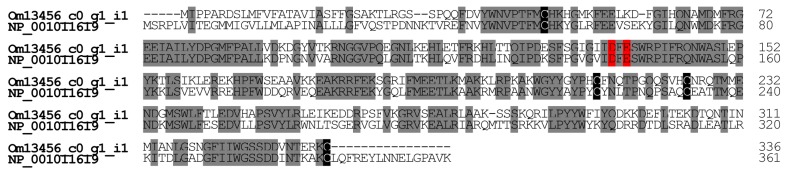
Alignment of hyaluronidase amino acid sequences. Using ClustalW, the amino acid sequences of hyaluronidases were aligned. Identical amino acid residues of hyaluronidases are highlighted in grey. Conserved cysteine and identical amino acid residues of hyaluronidases are highlighted in red and grey, respectively. The nucleotide sequence of *O. monticola* hyaluronidase has been assigned DDBJ/EMBL/GenBank Accession Numbers FX985505.

Hyaluronidase occurs in many venomous animals, including ants and bees. Hyaluronidase degrades hyaluronic acid, thereby facilitating the penetration of the venom components into various tissues. It is also an allergen in *A. mellifera* venom (Api m 2) [21]. In *O. monticola* venom glands, one contig (Om13456_c0_g1_i1) encodes hyaluronidase (0.21% of all toxin-like peptide and protein reads). Both *O. monticola* and *A. mellifera* hyaluronidase have four conserved cysteine residues. *O. monticola* hyaluronidase displays significant homology (52.8%) with the amino acid sequences of *A. mellifera* hyaluronidase NP_001011619 (Figure 6).

#### 2.2.5. ICK-Like Peptides

We found four contigs in the *O. monticola* transcript data which encode ICK-like peptides. Three of them have six conserved cysteine residues (Om253_c0_g2_i1, Om12753_c3_g1_i1, and Om17608_c0_g1_i1) and are known as short-type ICK-like peptides 1, 2, and 3, respectively. The other has eight conserved cysteine residues (Om11798_c3_g4_i1 with 290 reads) and is called a long-type ICK-like peptide. The transcription levels of all ICK-like peptides are low (<0.1% of all toxin-like peptide and protein reads). Short-type ICK-like peptides 1, 2, and 3 share amino acid sequences (20.4–76.9% homology) with a *D. quadriceps* ortholog (XP_014475205.1). The ICK-like peptides seem to be ubiquitous among all ant species. Although they have diverse biological functions like antimicrobial activity and ion channel blocking, they are still poorly understood [22].

**Figure 7:**
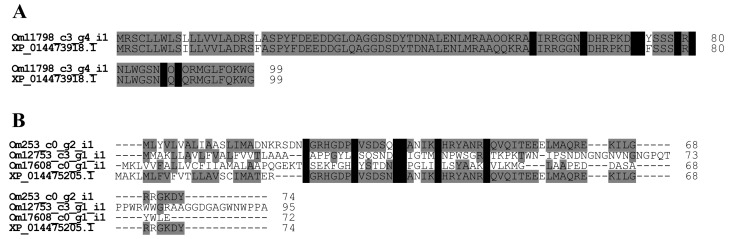
Multiple alignment alignment of ICK-like peptide amino acid sequences. Using ClustalW, the amino acid sequences of long- (**A**) and short- (**B**) type ICK-like peptides were aligned. Conserved cysteine and identical amino acid residues of ICK-like peptides are highlighted in black and grey, respectively. The nucleotide sequences of *O. monticola* ICK-like peptides have been assigned DDBJ/EMBL/GenBank Accession Numbers FX985500-985503.

The long-type ICK-like and the *D. quadriceps* ortholog (XP_014473918.1) have nearly identical amino acid sequences (96.9% identity) except for three amino acid substitutions at positions 10, 20, and 74 [4] (Figure 7).

#### 2.2.6. Dipeptidyl Peptidase 4

**Figure 8:**
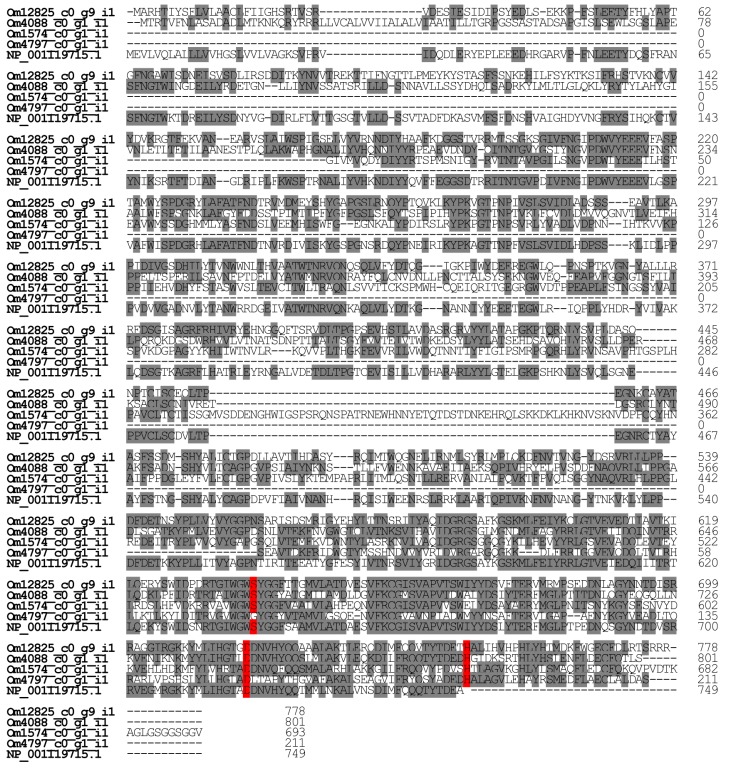
Multiple alignment of dipeptidyl peptidase amino acid sequences. Using ClustalW, the amino acid sequences of dipeptidyl peptidases were aligned. The key amino acid residues for enzymatic activity and identical amino acid residues were highlighted in red and grey, respectively. The nucleotide sequences of *O. monticola* dipeptidyl peptidases have been assigned DDBJ/EMBL/GenBank Accession Numbers FX985542-FX985545.

We found four contigs in the *O. monticola* transcript data encoding dipeptidyl peptidase 4 (Om12825_c0_g9_i1, Om4088_c0_g1_i1, Om1574_c0_g1_i1, and Om4797_c0_g1_i1). The dipeptyl peptidase 4 corresponding to Om12825_c0_g9_i1 constitutes 0.15% of all toxin-like peptide and protein reads in the venom gland. It displays significant homology with *D. quadriceps* venom dipeptidyl peptidase 4 isoform X2 (XP_014479089.1) (78.7%) and that of *A. mellifera* (NP_001119715.1) (53.4%). The other dipeptidyl peptidases 4, Om4088_c0_g1_i1, Om1574_c0_g1_i1, and Om4797_c0_g1_i1, closely resemble *D. quadriceps* dipeptidyl peptidase 4 isoform X2 (XP_014469652.1), *Harpegnathos saltator* dipeptidyl aminopeptidase C2E11.08 isoform X2 (XP_011146456.1), and *Harpegnathos saltator* dipeptidyl peptidase 4 (EFN87859.1) (94.3%, 93.2%, and 99.5%, respectively) (Figure 8). In *A. mellifera*, dipeptidyl peptidase 4 is a major venom allergen and is known as Api m 5. Despite the fact that *A. mellifera* dipeptidyl peptidase 4 is an allergen, *O. monticola* dipeptidyl peptidase 4 might serve to activate pilosulin-like peptides [15].

#### 2.2.7. Phospholipase A_2_-Like Proteins

As with the venom glands in other ant species, the phospholipase A_2_-like protein gene is expressed in those of *O. monticola.* We found five contigs encoding phospholipase A_2_-like proteins in *O. monticola*. Three of them (Om12710_c0_g1_i5, Om6159_c0_g1_i1, and Om13056_c3_g1_i1) significantly resemble (25.0–71.4%) the venom gland phospholipase A_2_ (XP_001120293.1 and XP_016768690.1) of *A. mellifera*, and two of them (Om13038_c1_g2_i11 and Om11043_c0_g2_i1) are similar to the housekeeping phospholipase ABHD3 and the calcium-independent phospholipase A_2_ gamma, respectively. The enzymatic activities of phospholipases A_2_ vary significantly with ant species [23]. The transcription levels of Om12710_c0_g1_i5, Om6159_c0_g1_i1, and Om13056_c3_g1_i1 were very low (<0.1% of all toxin-like peptide and protein reads), so the enzymatic activities of phospholipases A_2_ in the venom might be low as well.

**Figure 9:**
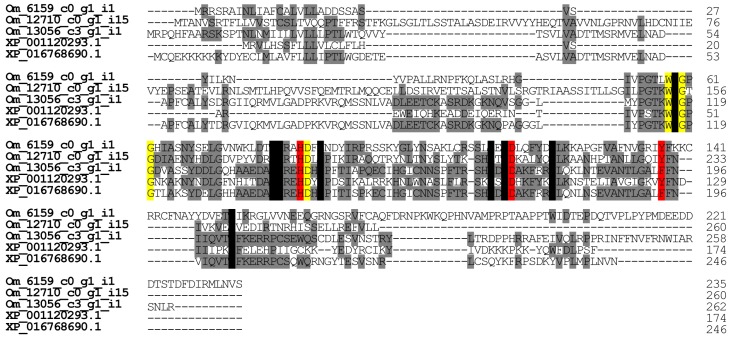
Multiple alignment of phospholipase A_2_-like protein amino acid sequences. Using ClustalW, the amino acid sequences of phospholipase A_2_-like proteins were aligned. Conserved cysteine and identical amino acid residues are highlighted of phospholipase A_2_-like proteins are highlighted in black and grey, respectively. The key amino acid residues for Ca^2+^ binding and enzymatic activity were highlighted in yellow and red, respectively. The nucleotide sequences of *O. monticola* phospholipase A_2_-like proteins have been assigned DDBJ/EMBL/GenBank Accession Numbers FX985506-FX985507.

Like other phospholipases A_2_, the *O. monticola* phospholipase A_2_-like protein has His^74^, Asp^104^, and Tyr/Phe^126^ which form a reactive center, Trp^48^, Gly^50^, Gly^52^, and Asp^75^ for Ca^2+^ binding, and eight conserved cysteine residues. (The numbering refers to XP_001120293.1) (Figure 9). Based on the phospholipase A_2_ classification by Dennis et al., *O. monticola* phospholipase A_2_-like protein might be a group III phospholipase A_2_ [24].

#### 2.2.8. Other Low-Abundance Transcripts of Toxin-Like Peptides and Proteins

The following transcripts were found for minor venom gland components: venom allergens 1–4 [25], three venom acid phosphatases, four venom serine proteases, two waprin-like peptides, one vascular endothelial cell growth factor (VEGF), one phospholipase D1, one Kazal protease inhibitor, one Kunitz protease inhibitor, six matrix metallopeptidases (MMPs), one secapin, one carboxypeptidase, and two carboxylesterase. The corresponding contig ID, accession numbers, lengths, read counts, identities with corresponding orthologs, and potential functions are summarized in Table S1. Most of these toxin-like proteins and peptides show significant homology with their corresponding orthologs in other ant species. Venom allergens 2 and 4, however, display low identities with the orthologs but both the proteins and their orthologs share 4 and 6 conserved cysteine residues, respectively.

### 2.3. Peptidomic Analysis

**Figure 10:**
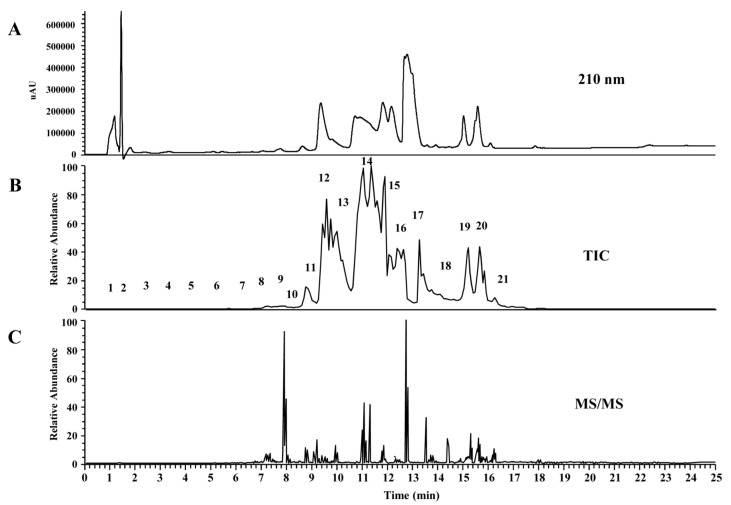
LC-ESI-MS (C18 column) profile of *O. monticola* venom extracts. Reverse-phase HPLC chromatogram pattern at A_210_ (**A**), the pattern of total ion current (*m*/*z* 100-2000) (**B**), and MS/MS spectrum (**C**) of *O. monticola* crude venom extracts were shown.

For MS/MS analysis of venom peptides, *O. monticola* venom was subjected to LC-MS by reverse-phase chromatography (C18 UG 120). On-line mass fingerprint was prepared from TIC by “virtual fractionation”, collecting MS spectra from a certain range of retention time (Figure 10). After MS/MS measurement in each fraction, we manually determined the sequence of the peptides. The obtained sequences were found to be truncated and intact pilosulin-like peptides by comparing with contigs from transcriptome analysis (Table 2 and Table S2). The possibility that pilosulin-like peptides are cleaved in the venom could not be excluded.

**Figure 11:**
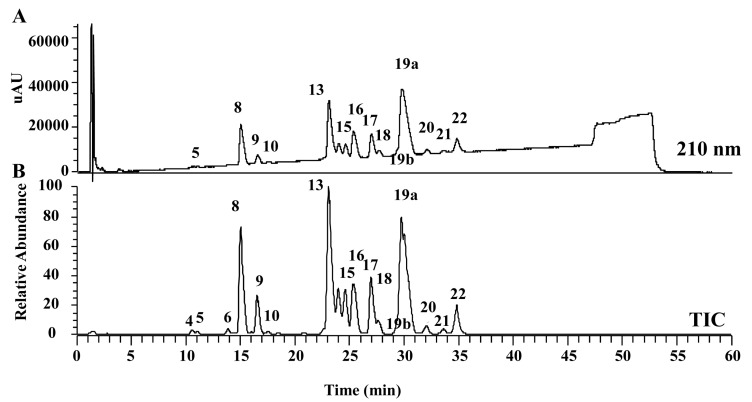
LC-ESI-MS (a wide pore C8 column) profile of *O. monticola* venom extracts. Reverse-phase HPLC chromatogram pattern at A_210_ (**A**) and the pattern of total ion current (*m*/*z* 100–2000) (**B**) of *O. monticola* crude venom extracts were shown.

A wide pore column (C8 SG300) gave better separation than a reverse-phase C18 column. The molecular masses in the major peak fractions were subjected to electrospray ionization-mass spectrometry (ESI-MS). The observed molecular masses were compared with the theoretical molecular mass of the pilosulin-like peptides predicted by transcriptome analysis (Figure 11). Some of the molecules eluted from the chromatography fractions could be assigned to pilosulin-like peptides 1–6 (Table 3). One major peak (27.4% of the total area) consisted of Om2061 molecule. The observed molecular mass of Om2061 molecule (2061.089) was identical to the theoretical molecular mass of pilosulin-like peptide 1. Two middle peaks (20.85% and 12.94%) consisted of Om6368 and Om4101 molecules, respectively.

The observed molecular mass of Om4101 (4101.244) was identical to the theoretical molecular mass of pilosulin-like peptide 3. Smaller peaks (7.78%, 6.97%, 5.42%, 5.27%, 4.29%, and 3.65% of the total area) consisted of Om3362, Om3325, Om6332, Om6350, Om3704, and Om1837 molecules, respectively. The observed molecular masses of Om3362 (3361.756), Om6332 (6331.631), Om3704 (3704.235), and Om1837 (1836.995) were nearly identical to the theoretical molecular masses of pilosulin-like peptides 2 (3361.753), 4 (6331.625), 6 (3704.231), and 5 (1837.071), respectively.

Comparing the mass profile of *O. monticola* with that of *O. hastatus* from a previous study, the proportion of the *O. monticola* molecules with a mass >5 kDa was higher than that of *O. hastatus.* We detected the molecules Om6368, Om6350, Om6332 and others in *O. monticola* venom.

We can obtain the preliminary amino acid sequence information from MS/MS analysis and partially correlate the peptidome data with that of the transcriptome. In future research, peptide sequencing by MS/MS with trypsin digestion or Edman degradation will be necessary to confirm all the aforementioned pilosulin-like peptide assignments and to identify those that are as yet unassigned.

## 3. Conclusions

In this study, we identified 92 toxin-like peptides and proteins from *O. monticola* using a transcriptomic analysis. In addition, we found intact and truncated pilosulin 1, 2, and 3 by a peptidomic analysis. The primary structures of all toxin-like peptides resemble their orthologs in other ant species. The bioactivities of the peptides and proteins may vary depending on the habitat and environment of ants. Certain toxin-like peptides and proteins in this study were tentatively classified as allergens and their primary bioactivities may be enzymatic, enzyme inhibition, and ion channel blockage. In future studies, we will characterize the biological functions of the toxin-like peptides and proteins in *O. monticola*.

## 4. Materials and Methods

### 4.1. Ants

One *O. monticola* colony was collected in Musashimurayama, Tokyo, Japan, on 1 July 2016. The species was morphologically identified.

### 4.2. Transcriptome Analysis

Total RNA was extracted from 115 venom glands with sacs. A cDNA library was prepared using TruSeq Stranded mRNA Library Prep Kit (Illumina, San Diego, CA, USA). The sequencing was performed by Hokkaido System Science (Sapporo, Japan) using HiSeq2500 (Illumina, San Diego, CA, USA) with paired-end reads (100 base pairs). The adapter and low-quality sequences were eliminated from these reads by Cutadapt v. 1.8.1. The de novo assemblies were run with Trinity v. 2.1.1 in DDBJ [26]. Highly homologous and duplicated contigs were integrated by cd-hit-est (parameters: –M 0, –d 0, –c 0.98). A BLASTX search against 551,987 UniProtKB [27] proteins was performed for gene annotation purposes. All input reads were mapped to the contigs and counted by Bowtie 2 to estimate the relative expression levels of the venom gland transcripts.

### 4.3. Pilosulin-Like Peptide 1 cDNA Cloning and Sequencing

Total RNA isolated from the ants was reverse-transcribed to cDNA and amplified by PCR with Ex Taq (Takara, Otsu, Japan). The oligonucleotide primers used were Pilo U1 (5′-ATGAAACCGTCGGGTATCAC-3′) corresponding to nucleotides (nt) 9–28 and PLP-1as (5′-ATGGTGTGATTGTTTCATCTA-3′) corresponding to nucleotides (nt) 261–281 of pilosulin-like peptide 2 (OM12875_c1_g1_i1). The amplified products corresponding to the original and 5′ side of the cDNAs were cloned into pCR 2.1-TOPO (Thermo Fisher Scientific, Waltham, MA, USA). All inserts were sequenced using a Model 3500 Genetic Analyzer (Thermo Fisher Scientific, Waltham, MA, USA).

### 4.4. Liquid Chromatography-Mass Spectrometry (LC-MS) Analysis

Twenty *O. monticola* venom sacs were collected and extracted with 50% acetonitrile containing 0.1% *v*/*v* trifluoroacetic acid (50 μL) for 2 h at 4 °C. The extract was passed through a 0.45-μm filter and successively diluted with the extraction solvent to a final concentration of 0.04 sacs/μL. This dilution was used for the LC-MS analysis. The LC conditions were: solvent A, 0.1% *v*/*v* aqueous formic acid; solvent B, 0.1% *v*/*v* formic acid in acetonitrile; 5–65% linear gradient of solvent B in solvent A at a flow rate of 200 μL/min; column, Capcell Pak C18 UG 120 (1.5 × 150 mm, Shiseido, Tokyo, Japan) and Capcell Pak C8 SG300 (1.5 × 150 mm, Shiseido, Tokyo, Japan); column temperature, 25 °C. The molecular weights of the ant peptides were verified by LTQ Orbitrap XL-ETD (Thermo Fisher Scientific, Waltham, MA, USA). The MS conditions were: ionization, electrospray in positive mode; ion spray voltage, 4.6 kV; capillary temperature, 350 °C; capillary and tube lens voltages, 19 V and 35 V, respectively; detector, an orbitrap at a resolution of 60,000 at *m*/*z* 400. The mass spectrometer was calibrated with polytyrosine and the resolution was usually 1–3 ppm after measurement. The mass-to-charge ratios for multiple charged ions were deconvoluted to give molecular masses. Peptide sequences were manually determined from MS/MS spectra and were confirmed by MS-Product in ProteinProspector program [28].

## Figures and Tables

**Table 1:** Summary of *O. monticola* venom gland transcriptome.

Total number of reads	44,087,058
Average base pairs	100 bp
Total number of contigs	49,639
(after cd-hit-est)	43,662
(-rRNA, -Bacterial transcripts, +Pilosulin-like peptide 1)	41,764
N50 contig size	1466 bp

**Table 2:** Pilosulin-like peptide sequences analyzed from MS/MS spectra.

Fraction No.	Retention Time (min)	Amino Acid Sequence	Observed Molecular Mass ^a^	Derivative
9	8.15	KVMDWLKDKM-NH_2_	1291.677	PLP1
11	8.55	VMDWLKDKM-NH_2_	1163.582	PLP1
11	8.75	AAANAAASEALSAMASQNE	1776.795	PLP3
12	9.08	GLLDWGK	787.423	PLP1
12	9.21	GWGSLFK	793.412	PLP2
12	9.40	GWGSLFKT	894.458	PLP2
13	9.54	GWGSLFKTVGKM	1309.685	PLP2
14	10.95	GWGSLFKTVGKMLAKAAVK	1991.142	PLP2
14	11.08	KTALEAAANAAASEALSAMASQNE	2319.098	PLP3
15	11.86	GLLDWGKKVMDWLKDKm-NH_2_	2077.083	PLP1
16	12.75	GLLDWGKKVMDWLKDKM-NH_2_	2061.090	PLP1

^a^ monoisotopic mass, L = either L or I; m = methionine S-oxide.

**Table 3:** LC-MS peaks and estimates of pilosulin-like peptides (PLPs) based on transcriptome analysis.

Peak	ID	Retention Time (min)	Area (%)	Observed Molecular Mass ^a^	Transcriptome Analysis Assisted Estimation of Mature Peptides			
					Possible Amino Acid Sequence	Molecular Formula	Theoretical Molecular Mass ^a^	Derivative
4	Om3264	10.58	0.41	3263.957				
5	Om4117	11.04	0.25	4117.238				
6	Om3489	13.84	0.46	3488.881				
7b	Om4229	14.45	<0.1	4229.334	KIKWGKIFKKGGKLIGKTALEAAANAAASEAISAMASQNEK	C_188_H_317_N_53_O_55_S	4229.336	PLP3
7a	Om3101	14.45	<0.1	3100.615				
8	Om4101	15.04	12.94	4101.244	KIKWGKIFKKGGKLIGKTALEAAANAAASEAISAMASQNE	C_182_H_305_N_51_O_54_S	4101.241	PLP3
9	Om3704	16.51	4.29	3704.235	IKGKKIMKNMGKAMKIAGKVAKAMAPIVVPLIVSAA-NH_2_	C_168_H_306_N_46_O_38_S_4_	3704.231	PLP6
10	Om4401	17.51	0.26	4401.364				
13	Om6368	23.07	20.85	6367.557				
14	Om6350	23.99	5.27	6349.589				
15	Om6332	24.62	5.42	6331.631	GVKELFGKAWGLVKKHLPKACGLLGYVKQ	C_300_H_484_N_78_O_68_S_2_	6331.625	PLP4
					|			
					GVKELFGKAWGLVKKHLPKACGLLGYVKQ			
16	Om3362	25.37	7.78	3361.756	GWGSIFKTVGKMIAKAAVKAAPEAISAMASQNE	C_149_H_224_N_40_O_44_S_2_	3361.753	PLP2
17	Om3325	26.94	6.97	3324.856				
18	Om2247	27.66	1.52	2247.188	GILDWGKKVMDWIKDKMGK	C_103_H_166_N_26_O_26_S_2_	2247.191	PLP1
19b	Om2119	29.26	<1.0	2119.094	GILDWGKKVMDWIKDKMG	C_97_H_154_N_24_O_25_S_2_	2119.096	PLP1
19a	Om2061	29.76	27.41	2061.089	GILDWGKKVMDWIKDKM-NH_2_	C_95_H_152_N_24_O_23_S_2_	2061.090	PLP1
20	Om1782	32.05	1.08	1782.019				
21	Om1812	33.62	0.67	1812.030				
22	Om1837	34.86	3.65	1836.995	IWGALLGTLIPAITSAIQ-NH_2_	C_87_H_144_N_20_O_23_	1837.071	PLP5

^a^ monoisotopic mass.

## Supplementary Materials

The following are available online at www.mdpi.com/2072-6651/9/10/323/s1, Figure S1: number of contigs derived from various bacterial genera, Table S1: other toxin-like peptides and proteins, Table S2: alignments of pilosulin-like peptide 1, 2, and 3 amino acid sequences from peptidomic and transcriptomic analyses.
