# Supplementary material for: Combined Venom Gland Transcriptomic and Venom Peptidomic Analysis of the Predatory Ant Odontomachus monticola
Source: Toxins (Basel). 2017 Oct 13;9(10):323. doi: 10.3390/toxins9100323 (PMC5666370; doi:10.3390/toxins9100323)
Supplement: Supplementary file 1 [file toxins-09-00323-s001.pdf]

Supplementary Materials: Combined Venom Gland Transcriptomic and Venom Peptidomic Analysis of the Predatory Ant *Odontomachus monticola*

Kohei Kazuma, Keiichi Masuko, Katsuhiro Konno and Hidetoshi Inagaki

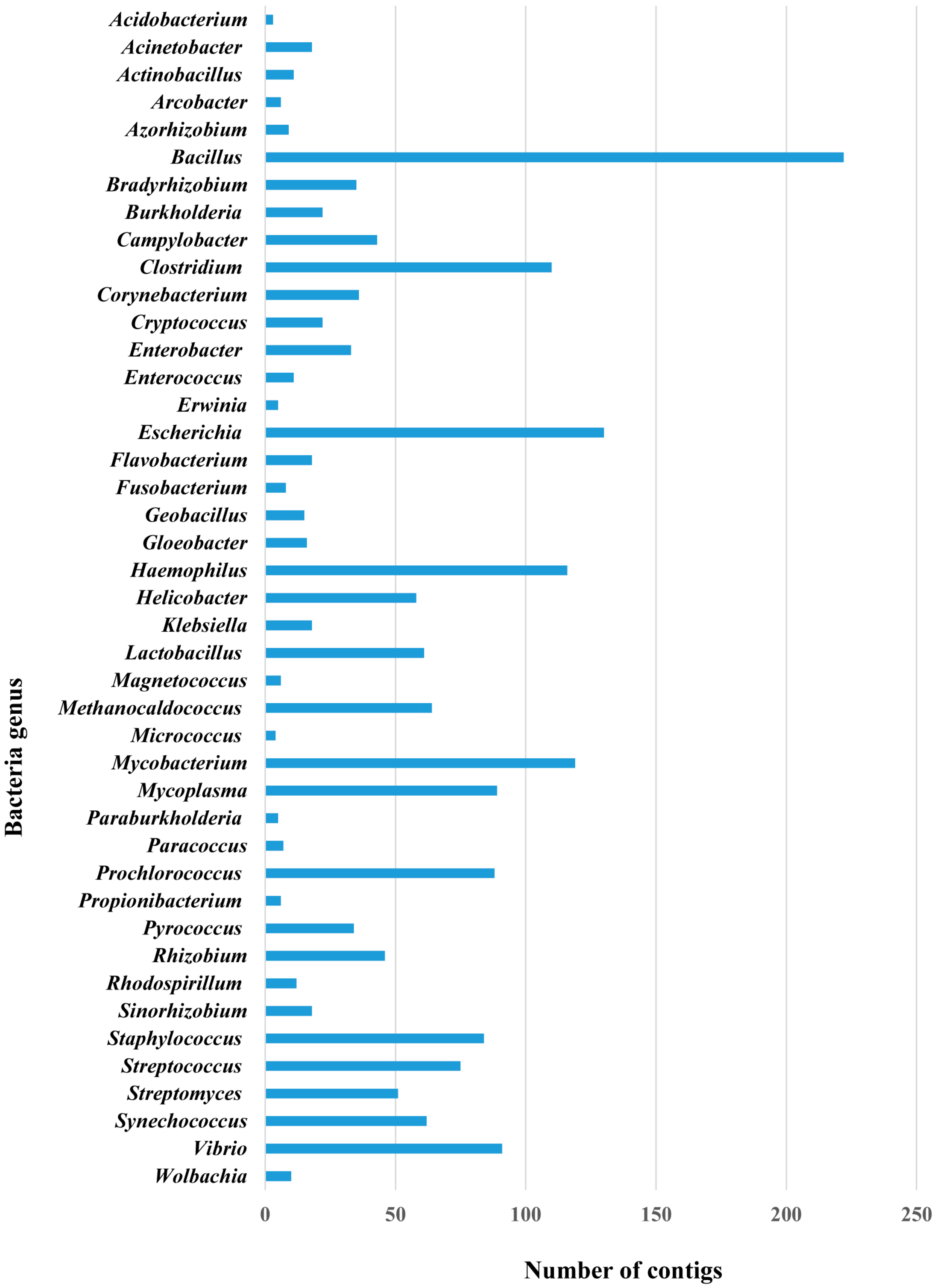

Figure S1. Number of contigs derived from various bacterial genera.

Table S1. Other toxin-like peptides and proteins.

The putative toxin name	Contig ID	ACC number	Length (bp)	Reads	Identity with corresponding ortholog	Potential function
Venom allergen 1 (PLA_1)	Om11857_c0_g1_i2	FX985511	1637	1668	*Ooceraea biroi* Phospholipase A_1 (EZA56721.1) 53.0%	Venom diffusion Tissue damage Allergy
Venom allergen 2	Om26417_c0_g1_i1	FX985512	707	48	*Solenopsis geminata* venom allergen 4 (Q9NH75.1) 26.9%	Allergy
					Dinoponera quadriceps uncharacterized protein (XP_014487550.1) 46.8%	
Venom allergen 3	Om7646_c0_g1_i1	FX985513	994	1945	*Dinoponera quadriceps* venom allergen 3-like (XP_014469499.1) 60.6%	Allergy
Venom allergen 4	Om27249_c0_g1_i1	FX985514	692	423	*Solenopsis invicta* Venom allergen 4 (XP_011172701.1) 24.8%	Allergy
					Dinoponera quadriceps uncharacterized protein (XP_014487550.1) 36.8%	
Venom acid phosphatase 1	Om3982_c1_g1_i1	FX985517	705	73	*Ooceraea biroi* Venom phosphatase A1 (EZA56721.1) 53.6%	Allergy
Venom acid phosphatase 2	Om13241_c0_g1_i2	FX985518	773	95	*Harpegnathos saltator* venom acid phosphatase Acph-1 (XP_011144396.1) 72.4%	
Venom acid phosphatase 3	Om8247_c0_g2_i1	FX985519	2526	1543	*Harpegnathos saltator* venom acid phosphatase Acph-1 (XP_011149152.2) 68.3%	
Venom serine protease 1	Om8957_c0_g1_i1	FX985522	1625	2434	*Harpegnathos saltator* venom serine protease Bi-VSP (XP_011142320.1) 82.4%	Venom diffusion Tissue damage
Venom serine protease 2	Om7503_c0_g1_i1	FX985523	1483	1416	*Dinoponera quadriceps* venom protease-like (XP_014477083.1) 62.8%	
Venom serine protease 3	Om13129_c3_g1_i3	FX985524	3750	810	*Harpegnathos saltator* serine protease snake-like (XP_011142690.1) 79.5%	
Venom serine protease 4	Om10669_c0_g1_i1	FX985525	3565	78	*Harpegnathos saltator* serine protease snake (EFN89361.1) 75.1%	
Waprin-like 1	Om9947_c0_g4_i1	FX985515	1034	852	*Dinoponera quadriceps* waprin-Thr1-like (XP_014487667.1) 85.6%	Not classified (Protease Inhibitor)
Waprin-like 2	Om5029_c0_g2_i1	FX985516	1486	573	*Dinoponera quadriceps* waprin-Phi1-like (XP_014487661.1) 87.3%	
VEGF	Om8718_c0_g1_i1	FX985521	3797	1781	*Habropoda laboriosa* VEGF-like protein (KOC69027.1) 48.4%	Venom diffusion
					Harpegnathos saltator uncharacterized protein (XP_019698417.1) 64.7%	
Phospholipase D1	Om8739_c0_g1_i2	FX985534	3359	536	*Harpegnathos saltator* phospholipase D1 (XP_019701186.1) 91.1%	Venom diffusion
Kazal Protease Inhibitor	Om10389_c0_g1_i1	FX985520	819	727	*Drosophila busckii* Kaz1-ORFB (ALC43924.1) 51.9%	Not classified (Protease Inhibitor)
					Harpegnathos saltator uncharacterized protein (XP_011137886.1) 77.1%	

Kunitz type protease inhibitor	Om26503_c0_g1_i1	FX985536	543	716	*Vollenhovia emeryi* kunitz-type serine protease inhibitor (XP_011873052.1) 52.5%	Not classified (Protease Inhibitor)
Venom metalloproteinase 1	Om13234_c6_g1_i8	FX985526	3368	142	*Harpegnathos saltator* ADAM with thrombospondin motifs (XP_011152782.1) 95.8%	Venom diffusion Tissue damage
Matrix metalloproteinase 2	Om13249_c2_g1_i2	FX985527	3610	105	*Harpegnathos saltator* MMP-2 (XP_011138596.1) 87.8%	
Matrix metalloproteinase 14	Om12910_c3_g1_i1	FX985528	4848	79	*Dinoponera quadriceps* MMP-14 isoform X3 (XP_014475594.1) 93.8%	
ADAM 12	Om12161_c0_g1_i2	FX985529	5791	126	*Harpegnathos saltator* ADAM 19 (XP_011140269.2) 86.6%	
ADAM TS 7	Om480_c0_g1_i1	FX985530	3684	127	*Dinoponera quadriceps* ADAM with thrombospondin motifs 7-like (XP_014473812.1) 94.2%	
ADAM1 10	Om10140_c0_g1_i2	FX985531	3355	113	*Harpegnathos saltato* ADAM 10 (XP_011153168.1) 96.4%	
Secapin	Om3630_c0_g1_i1	FX985535	994	465	*Camponotus floridanus* Secapin (AEM44798.1) 67.5%	Allergy
					Dinoponera quadriceps uncharacterized protein (XP_014488812.1) 70.6%	
Carboxypeptidase	Om9464_c0_g1_i1	FX985537	2025	2618	*Harpegnathos saltato* venom serine carboxypeptidase (XP_011143619.1) 84.1%	Allergy
Venom carboxylesterase 1	Om13001_c0_g1_i2	FX985532	2862	4017	*Harpegnathos saltato* venom carboxylesterase-6-like (XP_011149322.1) 78.2%	Allergy
Venom carboxylesterase 2	Om21361_c0_g1_i1	FX985533	1842	1457	*Harpegnathos saltato* venom Esterase FE4 (EFN85480.1) 76.0%	
					Dinoponera quadriceps venom carboxylesterase-6-like (XP_014477483.1) 70.9%	

Potential function was followed by classification of dos Santos *et al* [25]. *O. monticola* toxin-like peptides and their orthologues were aligned by the John-Hein method in Lasergene 12 (DNASTAR, Madison, WI, USA).

Table S2. Alignments of pilosulin-like peptide 1, 2, and 3 amino acid sequences from peptidomic and transcriptomic analyses.

Peptide Name	Retention time	Observed/Theoretical molecular mass	Amino acid sequence
	8.15	787.423	GLLDWGK
	8.55	1291.677	KVMDWLKDKM-NH_2
	9.08	1163.582	VMDWLKDKM-NH_2
	11.86	2077.083	GLLDWGKKVMDWLKDKm-NH_2
	12.75	2061.090	GLLDWGKKVMDWLKDKM-NH_2
PLP1		2061.090	GLLDWGKKVMDWLKDKM-NH_2
	9.21	793.412	GWGSLFK
	9.40	894.458	GWGSLFKT
	9.54	1309.685	GWGSLFKTVGKM
	10.95	1991.142	GWGSLFKTVGKMLAKAAVK
PLP2		3361.753	GWGSIFKTVGKMIAKAAVKAAPEAISAMASQNE
	8.75	1776.795	EAAANAAASEALSAMASQNE
	11.08	2319.098	KTALEAAANAAASEALSAMASQNE
PLP3		4229.336	KIKWGKIFKKGGKLIGKTALEAAANAAASEALSAMASQNEK
